# Esophageal Crohn's Disease Treated “Topically” with Swallowed Aerosolized Budesonide

**DOI:** 10.1155/2010/418769

**Published:** 2010-09-30

**Authors:** Petros Zezos, Georgios Kouklakis, Anastasia Oikonomou, Michail Pitiakoudis, Constantinos Simopoulos

**Affiliations:** ^1^Gastrointestinal Endoscopy Unit, University General Hospital of Alexandroupolis, Democritus University of Thrace, Dragana, 68100 Alexandroupolis, Greece; ^2^Department of Radiology, University General Hospital of Alexandroupolis, Democritus University of Thrace, Dragana, 68100 Alexandroupolis, Greece; ^3^2nd Department of Surgery, University General Hospital of Alexandroupolis, Democritus University of Thrace, Dragana, 68100 Alexandroupolis, Greece

## Abstract

Proximal Crohn's disease, involving the esophagus, the stomach, the duodenum, and the proximal jejunum, is uncommon. Treatment for proximal Crohn's disease is based on data derived from case series than from controlled trials. We present a case of Crohn's colitis with concomitant proximal esophagogastroduodenal involvement treated with conventional treatment plus swallowed aerosolized budesonide as a novel adjuvant topical treatment for the esophageal disease, and we review the treatment options for proximal Crohn's disease.

## 1. Introduction

Crohn's disease (CD) is a chronic inflammatory disease of unknown etiology characterized by a chronic, granulomatous, segmental transmural inflammation that may affect any part of the gastrointestinal (GI) tract. The ileum, the colon, the rectum, and the perianal region are the most frequent locations of disease involvement. Proximal Crohn's disease, involving the esophagus, the stomach, the duodenum and the proximal jejunum, is uncommon with a prevalence of 0.5% to 13% in patients with ileocolonic CD [1, 2], while isolated esophageal CD has been rarely described [3]. Although upper GI CD is uncommon in adults, it is seen frequently in children [3]. Patients with proximal CD usually present with concomitant small or large bowel disease. Today, the incidence of proximal disease is increased since CD patients more frequently undergo upper GI endoscopy. The recent European consensus (European Crohn's and Colitis Organization (ECCO)) on diagnosis and management of Crohn's disease propose treatments for esophageal and gastroduodenal disease based on data derived from case series than from controlled trials [4]. In this paper we present a case of Crohn's colitis with concomitant proximal esophago-gastroduodenal involvement that was treated with conventional treatment according to ECCO guidelines plus swallowed aerosolized budesonide as a novel adjuvant topical treatment for esophageal disease.

## 2. Case Presentation

**Figure 1:**
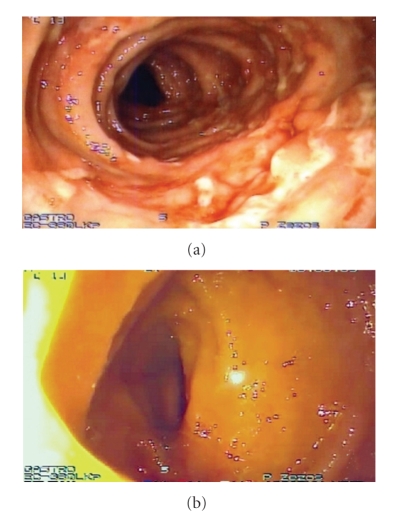
Colonoscopy demonstrated active colitis with longitudinal ulcers from sigmoid colon through cecum (a) and a normal terminal ileum (b).

A 21-year-old male presented with abdominal pain, watery diarrhea, low-grade fever and a 5 kg weight loss during the last 3 weeks. The patient was lean (Body Mass Index: 16.8 Kg/cm), did not smoke or drink alcohol. His past history was unremarkable and did not take any medication. Physical examination revealed mild diffuse abdominal tenderness. Laboratory studies revealed elevated ESR, CRP, platelet count, and the presence of leukocytes in the stool without blood, parasites, or ova. Chest X-ray was normal, and Mantoux test was negative. Colonoscopy showed longitudinal ulcers from sigmoid colon through cecum (Figure 1(a)) and a normal terminal ileum (Figure 1(b)). Histological examination demonstrated features of active Crohn's colitis without ileitis. Small bowel follow-through series were normal. Treatment with oral mesalazine (3 g/day) and metronidazole (500 mg twice daily for 10 days) was started. 

**Figure 2:**
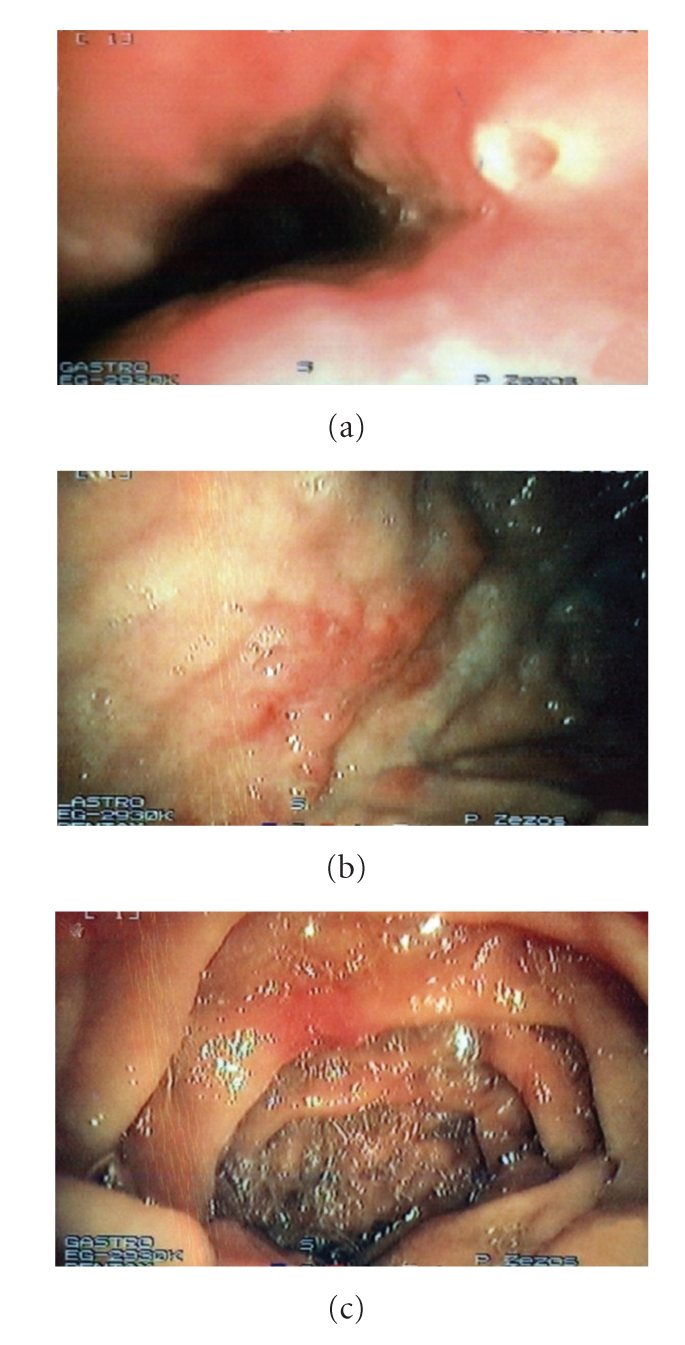
Upper gastrointestinal endoscopy revealed proximal Crohn's disease with erosions in esophagus (a), stomach (b) and duodenum (c).

The patient had no symptoms of proximal disease, but it is the standard practice in our department to perform supplementary upper GI endoscopy in all patients with ileal and/or colonic CD in order to define the sites, the extent, and the phenotype of the disease according to Montreal classification [4]. Gastroscopy revealed multiple punch-out ulcers in the esophagus (Figure 2(a)) and erosions in the stomach (Figure 2(b)) and the duodenum (Figure 2(c)). Although histology showed nonspecific mild inflammatory reaction without evidence of cytomegalovirus (CMV), herpes simplex virus (HSV), or Candida albicans infection, the endoscopic appearance, the location and extent of the lesions combined with the active Crohn's colitis and the negative history of NSAIDs use, were suggestive for proximal CD. 

**Figure 3:**
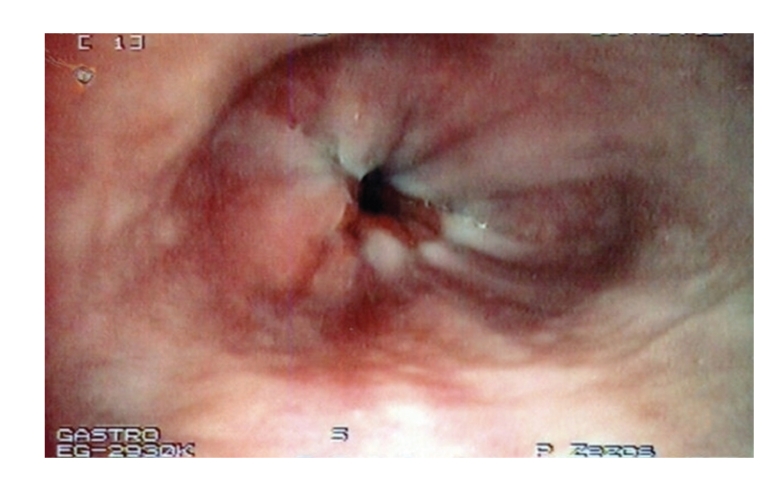
Follow-up with upper gastrointestinal endoscopy showing complete healing of the esophageal erosions after 3 months of topical treatment with swallowed budesonide.

Getting the idea from eosinophilic esophagitis' treatment [5, 6], “topical” treatment with ingestion of inhalable budesonide (Pulmicort turbuhaler; AstraZeneca LP, Sweden) together with oral pantopazole were added for the management of the esophageal and the gastroduodenal disease. The symptoms of colitis subsided completely after 2 weeks and in the 3rd month of followup the patient reported a 10-kg weight gain. A followup upper GI endoscopy showed disappearance of the esophageal disease (Figure 3) with persistence of gastric and duodenal erosions. Since the patient was asymptomatic from the proximal and distal disease he was set in a regular followup schedule with the same treatment (5-ASA, (PPIs) and inhalable budesonide) and the recommendation to avoid the use of NSAIDs. 

 Unfortunately, a few months later the patient experienced a new flare of the distal disease, which was managed successfully with systemic corticosteroids, starting with 32 mg oral methyl-prednisolone and following a 12-week tapering scale after remission of symptoms was noted. Subsequently, azathioprine (2 mg/kg/day) was added to the treatment as a steroid-sparing agent to maintain remission. Complete and sustained disease remission, clinical and endoscopic, was achieved throughout the gastrointestinal tract as evidenced during followup visits.

## 3. Discussion

Usually the proximal involvement is diagnosed when a CD patient with known small and/or large bowel disease undergoes endoscopy for complaints or symptoms mimicking upper GI diseases, including gastroesophageal reflux disease, dyspepsia, and peptic ulcer disease. Sometimes the proximal involvement is silent and the disease is diagnosed incidentally during endoscopy in an asymptomatic patient [1, 3].

Upper GI endoscopy with multiple mucosal biopsies will establish the diagnosis of proximal Crohn's disease in most of the cases. In the majority of cases the proximal disease extends beyond the esophagus, involving the stomach and the duodenum too. The most common endoscopic features in esophageal Crohn's disease include single or multiple erosions, aphthous ulcers or deep punched-out ulcers, with either local or extensive involvement surrounded by normal-looking mucosa. The antrum and the duodenum are most frequently involved than the proximal stomach is. Granular mucosa, patchy erythema, superficial or deep ulcerations can be observed [1]. Stenosis is the major complication of proximal CD while fistulas are infrequent [1].

The lesions in the proximal involvement must be differentiated from diseases affecting the organ involved. Lesions in the esophagus must be differentiated from reflux esophagitis and infectious esophagitis (CMV, HSV, Candida albicans). Helicobacter pylori gastritis and granulomatous gastritis (Wegener's granulomatosis or necrotizing vasculitis) must be excluded in the stomach. Giardiasis, coeliac disease, lymphoma must be considered in the diagnosis when duodenum is involved. Of course when features of CD are present in the distal GI tract the diagnosis is more secure [1].

 Treatment of proximal CD depends on the severity of the disease. The ECCO consensus underscores the lack controlled studies to demonstrate the effectiveness of drug therapy and proposes the addition of a proton pump inhibitor in the conventional treatment for distal disease or the use of systemtic corticosteroids and thiopurines or methotrexate [4]. The administration of 5-aminosalicylates (5-ASA) is a logical treatment, but their effect is not certain since these products are not activated in the proximal gastrointestinal tract. Antisecretory drugs (histamine receptor antagonists; HRAs and proton-pump inhibitors; PPIs) may relieve pain, but their efficacy in mucosal healing has not been proven [1]. On the other hand, some authors raised the concern that omeprazole monotherapy could lead to stricturing and fistulization because of continued inflammation [3, 7]. Corticosteroids as a systemic therapy are the best treatment of choice, while no data exist on the therapeutic effect of budesonide formulations [1]. Immunosuppressive therapy with azathioprine, 6-mercaptopurine or methotrexate is an excellent steroid-sparing option for long-term maintenance which is also indicated for the management of the concomitant distal disease [2, 4]. Anti-TNF therapy is an alternative treatment for severe or refractory disease as recommended by ECCO consensus which suggested that clinicians should have a lower threshold for starting anti-TNF therapy than for disease elsewhere, given the poor prognosis of Crohn's disease with proximal involvement [4, 8, 9].

 There is no recommendation for topical treatment of proximal CD with steroids or 5-ASA, although Rholl et al. reported the successful management of esophageal Crohn's disease complicated with an esophagogastric fistula with topical administration of 5-ASA and steroids in liquid form [10]. 

 In our case we used aerosolized budesonide indicated for asthma, instructing the patient not to inhale but to swallow the drug as has been suggested in eosinophilic esophagitis treatment [5, 6]. With this application we consider that a sufficient amount of budesonide will reach the entire esophagus, achieving in that way a “topical” treatment. Indeed, in a followup endoscopy the esophageal lesions were healed, but the more distal disease was still active. Finally, the addition of azathioprine induced and maintained remission was curative for all locations of disease.

## 4. Conclusion

We believe that routine upper GI endoscopy in patients with CD, even when topical symptoms are lacking, helps to diagnose proximal involvement which must be differentiated from other ulcerative conditions [11, 12]. The optimal treatment for esophageal CD has not been established but topical corticosteroids offer an effective adjuvant therapeutic option together with those classically suggested for proximal disease [2].
